# Circadian factors BMAL1 and RORα control HIF-1α transcriptional activity in nucleus pulposus cells: implications in maintenance of intervertebral disc health

**DOI:** 10.18632/oncotarget.8521

**Published:** 2016-03-31

**Authors:** Kaori Suyama, Elizabeth S. Silagi, Hyowon Choi, Kou Sakabe, Joji Mochida, Irving M. Shapiro, Makarand V. Risbud

**Affiliations:** ^1^ Department of Orthopaedic Surgery, Sidney Kimmel Medical College, Thomas Jefferson University, Philadelphia, USA; ^2^ Department of Orthopaedic Surgery, Surgical Science, Tokai University School of Medicine, Isehara, Kanagawa, Japan; ^3^ Department of Anatomy and Cellular Biology, Basic Medical Science, Tokai University School of Medicine, Isehara, Kanagawa, Japan; ^4^ Program in Cell and Developmental Biology, Jefferson College of Biomedical Sciences, Thomas Jefferson University, Philadelphia, USA

**Keywords:** intervertebral disc, nucleus pulposus, HIF-1, BMAL1, RORα, Pathology Section

## Abstract

BMAL1 and RORα are major regulators of the circadian molecular oscillator. Since previous work in other cell types has shown cross talk between circadian rhythm genes and hypoxic signaling, we investigated the role of BMAL1 and RORα in controlling HIF-1-dependent transcriptional responses in NP cells that exist in the physiologically hypoxic intervertebral disc. HIF-1-dependent HRE reporter activity was further promoted by co-transfection with either BMAL1 or RORα. In addition, stable silencing of BMAL1 or inhibition of RORα activity resulted in decreased HRE activation. Inhibition of RORα also modulated HIF1α-TAD activity. Interestingly, immunoprecipitation studies showed no evidence of BMAL1, CLOCK or RORα binding to HIF-1α in NP cells. Noteworthy, stable silencing of BMAL1 as well as inhibition of RORα decreased expression of select HIF-1 target genes including VEGF, PFKFB3 and Eno1. To delineate if BMAL1 plays a role in maintenance of disc health, we studied the spinal phenotype of BMAL1-null mice. The lumbar discs of null mice evidenced decreased height, and several parameters associated with vertebral trabecular bone quality were also affected in nulls. In addition, null animals showed a higher ratio of cells to matrix in NP tissue and hyperplasia of the annulus fibrosus. Taken together, our results indicate that BMAL1 and RORα form a regulatory loop in the NP and control HIF-1 activity without direct interaction. Importantly, activities of these circadian rhythm molecules may play a role in the adaptation of NP cells to their unique niche.

## INTRODUCTION

The intervertebral disc is a complex tissue that permits a range of motion between adjacent vertebrae and accommodates high biomechanical forces in the spine. It consists of an outer fibrocartilaginous annulus fibrosus (AF) that encloses a gel-like nucleus pulposus (NP). Intervertebral discs exhibit diurnal changes in height and composition. This is caused by high compressive loading on the disc throughout the day that results in efflux of fluid from the tissues. At night, under low loading conditions, osmotic pressure provides a driving force to imbibe fluid into the disc [1–3]. Several studies have shown that NP cells respond to changes in osmotic loading by altering their gene expression and anabolic activities [4–6]. Noteworthy, NP tissue is also completely avascular and thus cells are adapted to survive in a hypoxic environment [7–12]. Previous reports clearly show that HIF-1α is a critical factor for NP cell survival [13] and drives their glycolytic metabolism [7–9]. Our group has previously shown that HIF-1α regulation in NP cells is unique. Particularly, there is robust HIF-1α expression even under normoxic conditions and its expression is not appreciably induced under hypoxia [7–12].

BMAL1 (brain and muscle ARNT-like1) and CLOCK are basic helix-loop-helix-PAS (bHLH-PAS) transcriptional factors and major components of the circadian molecular oscillator [14–16]. BMAL1 and CLOCK form a heterodimer and, by binding to E-box motifs in promoters, stimulate transcription and translation of cryptochrome (CRY) and period (PER) family members. In turn, these genes inhibit BMAL1:CLOCK activity through formation of repressive complexes [17, 18]. Another important regulatory loop that controls circadian clock is made up of BMAL1, Retinoic acid receptor-related Orphan Receptor (ROR)-α and REV-ERBα, a transcriptional repressor [15, 19, 20]. RORα and REV-ERBα competitively bind ROR binding sequences (RORE) in the BMAL1 promoter controlling its levels; meanwhile, RORα and REV-ERBα transcript levels are controlled by BMAL1:CLOCK [15, 16, 21]. HIF-α and HIF-β (ARNT) proteins also belong to the mammalian bHLH-PAS family [22]. The function of BMAL1 and RORα in controlling hypoxic HIF-1 activity through protein-protein interaction has been reported in certain cell types [23–26] indicating that BMAL1 and RORα may promote HIF-1α target gene expression. However, whether a relationship between HIF-1α and circadian regulators BMAL1 and RORα exists in NP cells is not known.

In this paper, we use *in vitro* and *in vivo* approaches to test the hypotheses that BMAL1 and RORα control hypoxia and HIF-1- dependent transcriptional responses in NP cells, and dysregulation of BMAL1 would compromise disc health. We show here, for the first time, that BMAL1 and RORα modulate HIF-1α transcriptional activity and influence HIF-1α target genes expression in NP cells. Moreover, *in vivo* studies using BMAL1 null mice suggest that BMAL1 deficiency may alter disc structure and function. Taken together, our findings suggest that both BMAL1 and RORα are important regulators of NP cell function.

## RESULTS

### Expression analysis of BMAL1 and other related factors in NP cells

**Figure 1:**
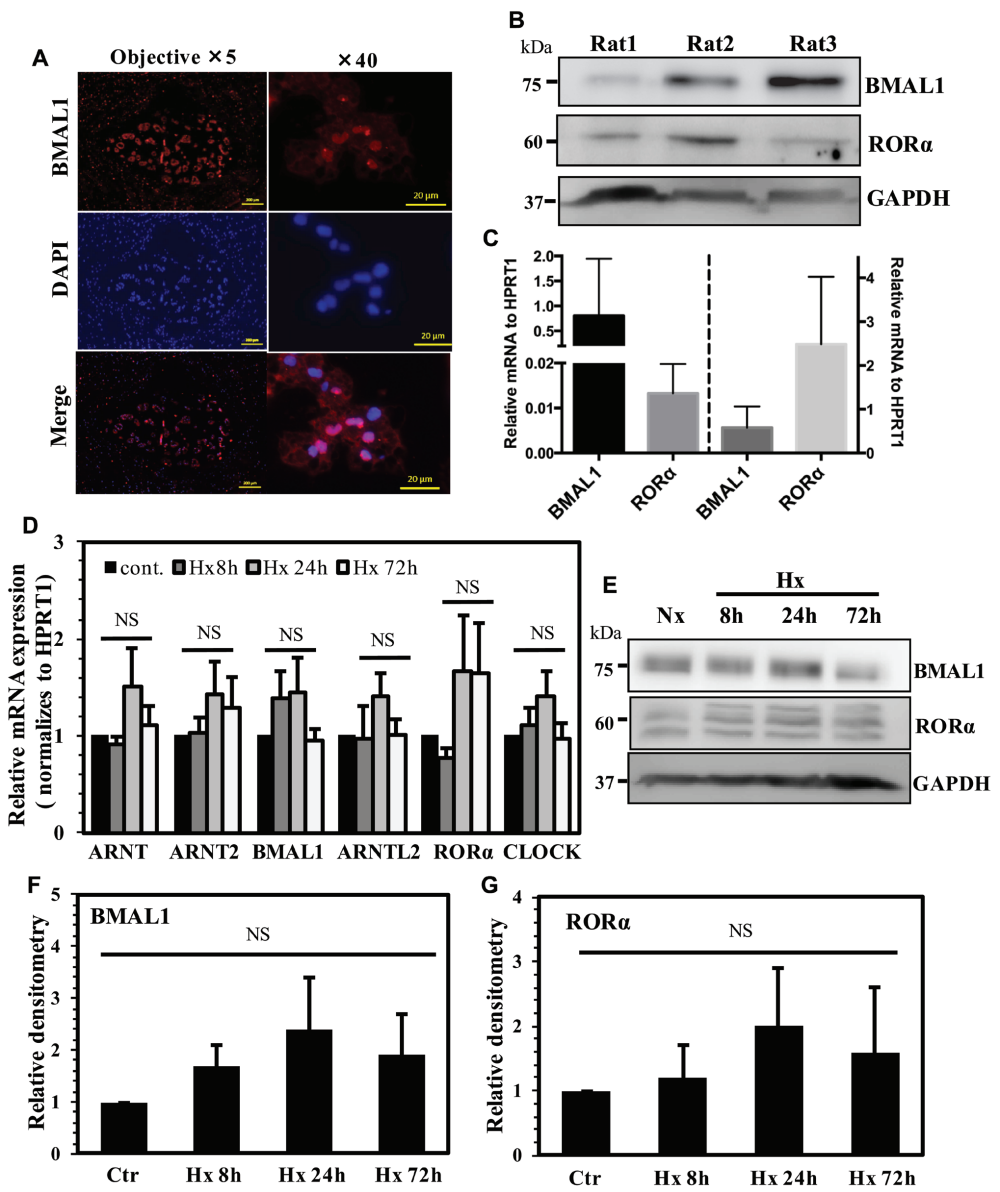
Expression analysis of BMAL1 and other related factors in NP cells **A**. Immunohistochemical localization of BMAL1 in rat intervertebral disc. Sagittal sections of the mature rat intervertebral disc, immunostained with BMAL1 antibody, showed prominent nuclear expression in NP tissue. **B**. Western blot analysis of BMAL1 and RORα expression in NP tissues isolated from three rats showed positive expression for both the proteins. **C**. qRT-PCR analysis of BMAL-1 and RORα mRNA expression from NP and AF tissues from rat discs (n=3 animals/group) **D**. qRT-PCR analysis of BMAL1, RORα, ARNT, ARNT2, ARNTL2 and CLOCK expression in rat NP cells cultured under hypoxia (1% O_2_). None of the genes showed significant increase in hypoxia. **E**. Western blot analysis of BMAL1 and RORα in NP cells cultured under hypoxia. **F**., **G**. Densitometric analysis of multiple blots shown in (E) above. No significant differences were seen between normoxic and hypoxic levels of BMAL1 and RORα. Data is represented as mean ± SE, n=3, p<0.05.

To investigate expression of BMAL1 in the intervertebral disc, we stained sections of rat discs with antibodies against BMAL1 (Figure 1A). The results show prominent expression of BMAL1 in NP tissue with many cells evidencing nuclear localization. Western blot was used to analyze the presence of BMAL1 and RORα proteins in NP tissues isolated from 3 rats. The expression of both BMAL1 and RORα was evident in NP tissue (Figure 1B). In addition, we measured mRNA expression of BMAL1 and RORα in NP and AF compartments of the disc. Both tissues indeed expressed BMAL1 and RORα transcripts (Figure 1C). To evaluate the effect of hypoxia on expression of BMAL1 and other ARNT family members, as well as important circadian rhythm genes, we measured mRNA and protein expression in NP cells cultured under hypoxia using qRT-PCR (Figure 1D) and Western blot analysis (Figure 1E). Our results show that mRNA expression of ARNT (HIF-1β), ARNT2, BMAL1, ARNTL2, RORα and CLOCK did not significantly change under hypoxia (Figure 1D). While there was a trend of increased protein levels of BMAL1 and RORα under hypoxia, it failed to reach statistical significance (Figure 1F, 1G).

### BMAL1 synergizes HIF-1 dependent HRE activity in NP cells

**Figure 2:**
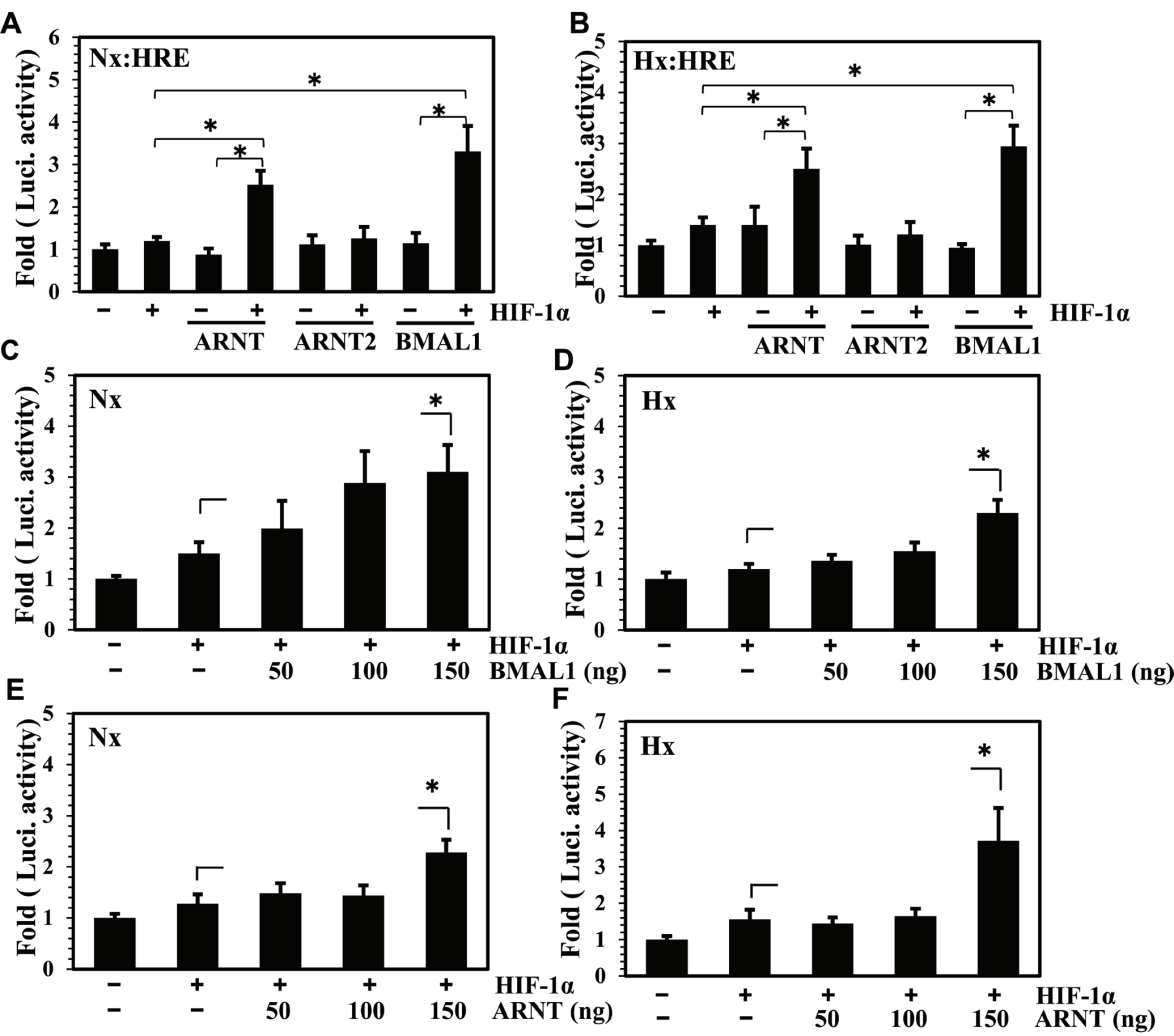
BMAL1 synergizes HIF-1 dependent HRE activity in NP cells **A**., **B**. Evaluation of HRE activity in NP cells transfected with ARNT, ARNT2, and BMAL1 (150 ng/each) with or without 25 ng of HIF-1α following 24 h culture in hypoxia or normoxia. Co-transfection of BMAL1 and ARNT but not ARNT2 with HIF-1α significantly increased the activity of HRE compared to HIF-1α alone. **C**.-**F**. Evaluation of HRE activity in NP cells co-transfected with increasing dose of BMAL1(C, D) or ARNT (E, F) with 25 ng of HIF-1α, after 24 h hypoxia and normoxia treatment. The HRE activity showed a significant increase at highest dose of BMAL1 and ARNT compared to HIF-1 alone. Data is represented as mean ± SE, n=3, * p<0.05.

We evaluated the effect of BMAL1 on activity of a HIF-responsive luciferase reporter (HRE-Luc). Co-transfection of BMAL1 with a low dose of HIF-1α promoted HIF-1 mediated activation of the HRE reporter under both normoxia and hypoxia (Figure 2A and 2B). A similar increase in activity was seen when ARNT, but not ARNT2, was co-transfected with HIF-1α (Figure 2A and 2B). However, addition of BMAL1 or ARNT alone had little effect on HRE activity. We then measured dose-dependency of BMAL1 or ARNT on HRE reporter activity driven by a sub optimal dose of HIF-1 (Figure 2C-2F). There was a trend of increasing HRE activity when 50 ng and 100 ng of BMAL1 or ARNT were co-transfected with HIF-1α, however a significant change in HRE activity was noted when BMAL1 (Figure 2C and 2D) and ARNT (Figure 2E and 2F) were used at 150 ng. All results were compared with HIF-1α treatment alone.

### BMAL1 controls HIF-1 activity without affecting HIF-α-TAD function

**Figure 3:**
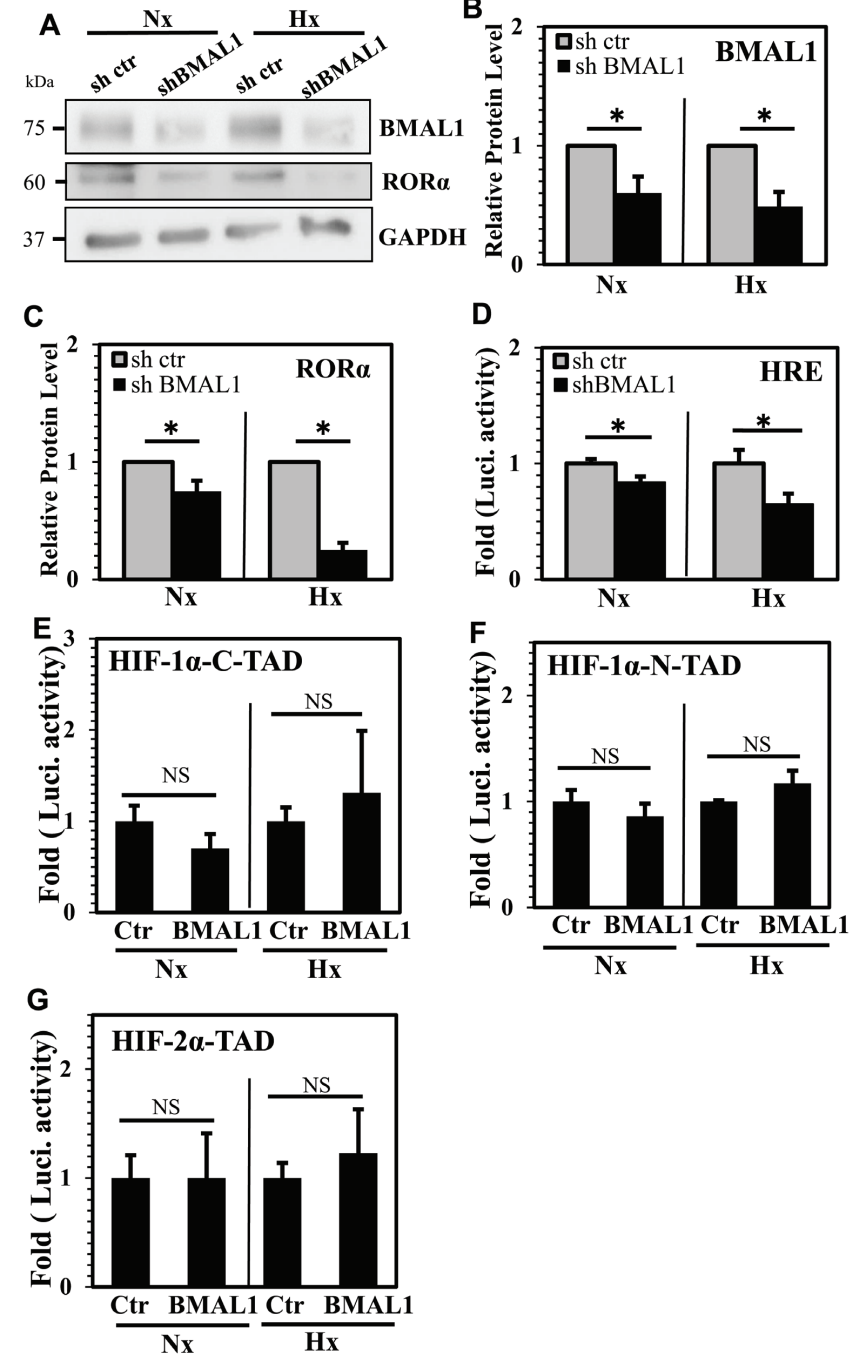
BMAL1 controls HIF-1 activity without affecting HIF-α-TAD function **A**. Evaluation of BMAL1 and RORα expression by Western blot in NP cells stably transduced with lentivirus expressing BMAL1 shRNA. **B**., **C**. Densitometric analysis of multiple blots shown in (A) demonstrated decreased BMAL1 (B) and RORα (C) expression in BMAL1-silenced cells under both normoxia and hypoxia. **D**. HRE activity of BMAL1-silenced NP cells is significantly lower than cells transduced with control shRNA. **E**.-**G**. Evaluation of BMAL1 control of HIF-α-TAD function. BMAL1 overexpression had no effects on HIF-1α-C-TAD (E), HIF-1α-N-TAD (F), as well as HIF-2α-TAD (G) regardless of oxygen tension. Data is represented as mean ± SE, n= 3, * p<0.05.

Due to the previously reported contribution of BMAL1 in controlling HIF-1 and HIF-2 function in other cell types [23–25, 27], we evaluated mechanisms by which BMAL1 controlled HIF-1 activity in NP cells. First, we stably knocked down BMAL1 in rat NP cells via transduction of lentiviral shRNA. Western blot and densitometric analysis confirmed that protein levels of BMAL1 and RORα, a known target of BMAL1, were lower in BMAL1-silenced NP cells under both hypoxia and normoxia (Figure 3A-3C). Moreover, activity of HIF-1α, as measured by HRE reporter, was significantly suppressed in BMAL1-silenced NP cells (Figure 3D). To determine if BMAL1 controlled HIF-α-TAD function, we measured TAD activity following BMAL1 overexpression. Results clearly showed that overexpression of BMAL1 did not result in significant change in activity of HIF-1α-C-TAD (Figure 3E), HIF-1α-N-TAD (Figure 3F), as well as HIF-2α-TAD (Figure 3G) regardless of oxygen tension.

### RORα controls HIF-1 transcriptional activity and TAD function in NP cells

**Figure 4:**
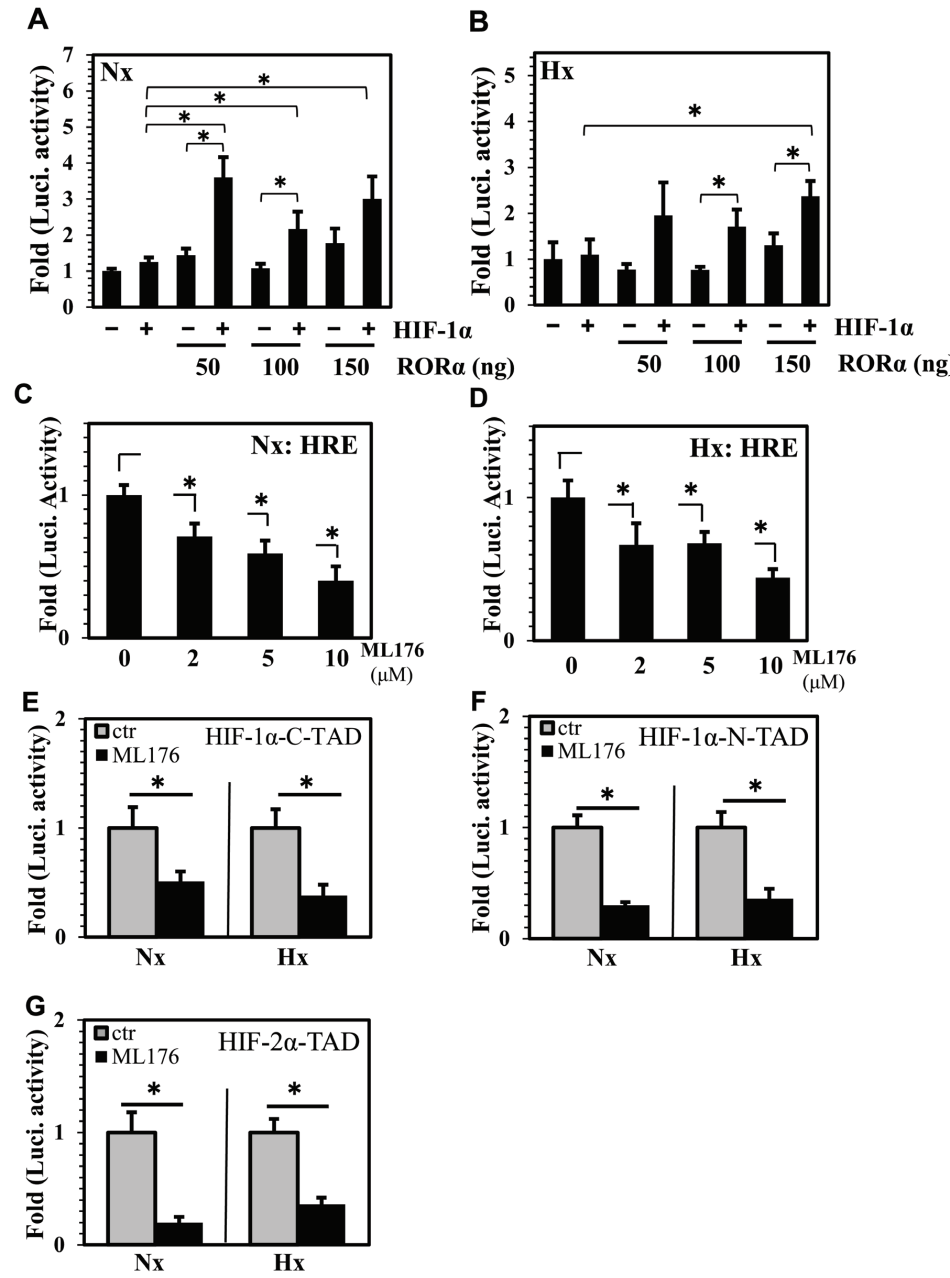
RORα controls HIF transcriptional activity and TAD function in NP cells **A.**, **B**. Evaluation of HRE activity in NP cells transfected with 50-150 ng of RORα with or without 25 ng of HIF-1α, after 24 h hypoxia and normoxia treatment. Under normoxia, compared to HIF-1α alone, cells co-transfected with RORα and HIF-1α showed a significant increase in HRE activity at all doses, while under hypoxia this increase was seen at 150 ng of RORα. **C**., **D**. Treatment of NP cells with increasing dose of highly specific, small molecular RORα inhibitor, ML176 (2-10 μM), resulted in decrease in activity of endogenous HIF protein under both normoxia (C) and hypoxia (D). **E**.-**G**. Regulation of HIF-α-TAD function by RORα in NP cells. Treatment with ML176 (10 μM) under both normoxia and hypoxia resulted in decrease in HIF-1α-C-TAD (E), HIF-1α-N-TAD (F) as well as HIF-2α-TAD (G) activities. Data is represented as mean ± SE, n=3, *p<0.05.

Since we observed that RORα expression was under the control of BMAL1 in NP cells, we evaluated the effect of RORα on HIF activity. When co-transfected under normoxia, there was a synergistic effect between RORα (50-150 ng) and HIF-1α on HRE reporter activity as compared to HIF-1α alone (Figure 4A). On the other hand, while a trend of increasing activity was seen in hypoxia at 50 and 100 ng of RORα, a significant synergistic increase in HIF-1-dependent HRE activity was only noted at the highest dose of 150 ng (Figure 4B). Importantly, regardless of the oxemic tension, RORα alone did not increase HRE activity at any of the doses tested.

To follow the RORα gain-of-function experiments, we performed loss-of-function experiments to delineate effects of RORα inhibition on HIF activity. For this purpose, we employed a highly specific, cell permeable, small molecular RORα activity inhibitor ML176 [28, 29]. Treatment of NP cells under both normoxia and hypoxia with increasing dose of ML176 (2-10 μM) resulted in a robust decrease in endogenous HIF activity at all doses as measured by HRE reporter activity (Figure 4C, 4D). We then measured HIF-α-TAD function in NP cells treated with ML176. Interestingly, inhibitor treatment resulted in a decrease in HIF-1α-C-TAD (Figure 4E), HIF-1α-N-TAD (Figure 4F) as well as HIF-2α-TAD (Figure 4G) activities, suggesting that RORα may affect HIF-1α transactivation.

### BMAL1 and RORα do not bind to HIF-1α in NP cells

**Figure 5:**
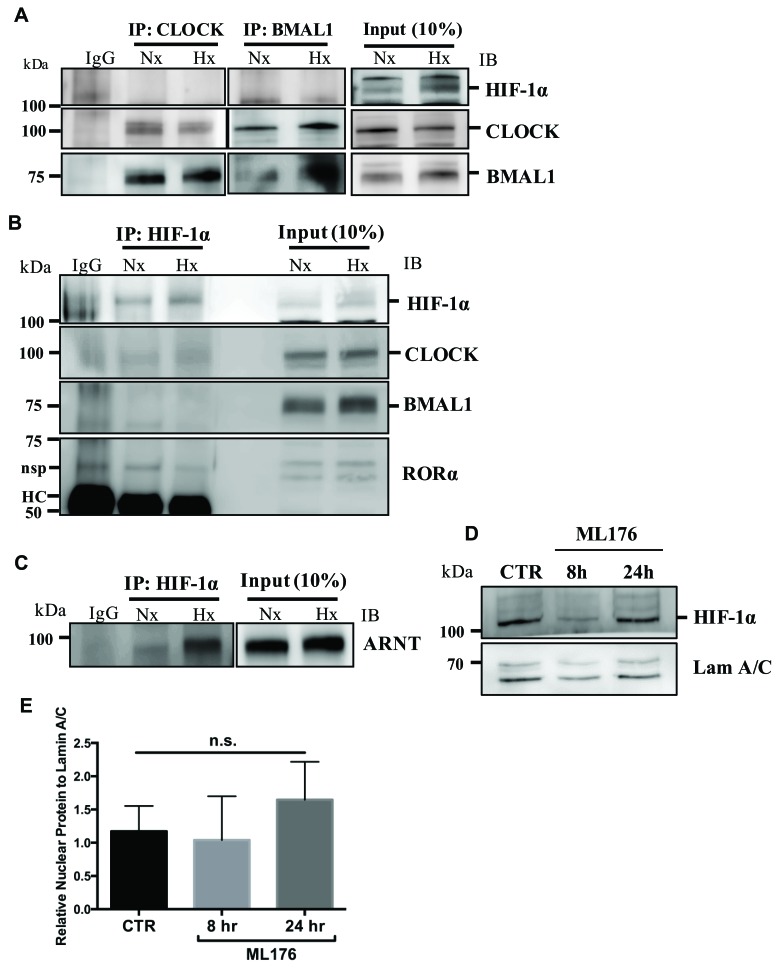
BMAL1 and RORα do not bind to HIF-1α in NP cells **A**. Immunoprecipitation (IP) of BMAL1 and CLOCK from NP cells cultured under normoxia or hypoxia for 24 h followed by Western blot analysis using anti-HIF-1α, anti-BMAL1 and anti-CLOCK antibodies. BMAL1 bound to CLOCK, but neither BMAL1 nor CLOCK bound to HIF-1α irrespective of oxygen tension. Preimmune rabbit IgG was used as a negative control for IP assays. **B**. Pulldown of HIF-1α did not show co-precipitation of BMAL1, CLOCK, or RORα. HC: heavy chain of IgG, nsp: non-specific (**C**) Pulldown of HIF-1α showed co- precipitation of HIF-1β/ARNT, association was higher under hypoxia. **D**., **E**. Treatment of NP cells with RORα inhibitor, ML-176 (10 μM), showed no effect on nuclear levels of HIF-1α. Densitometric analysis shown in (E) was performed on blots from 3 independent experiments, Data is represented as mean ± SE, * p < 0.05.

Given that BMAL1 and RORα control HIF-1α activity and function, we investigated the possibility of direct protein-protein interaction between BMAL1, RORα and HIF-1α. Surprisingly, immunoprecipitation of BMAL1 as well as CLOCK in NP cells did not result in pulldown of HIF-1α (Figure 5A). As expected, BMAL1 evidenced binding with CLOCK and immunoprecipitation of CLOCK resulted in co-precipitation of BMAL1 (Figure 5A). We also performed immunoprecipiation of HIF-1α to confirm these findings (Figure 5B). In line with BMAL1 and CLOCK IP results, we were unable to detect co-precipitation of BMAL1, CLOCK or RORα following pulldown of HIF-1α (Figure 5B). Again, HIF-1α was seen to associate with HIF-1β/ARNT (Figure 5C). These findings suggest that BMAL1 and RORα may not bind to HIF-1α or that the binding is highly transient. In order to determine if RORα affects HIF-1α levels and translocation in NP cells, we measured nuclear levels of HIF-1α following RORα inhibition under hypoxia. Western blot and corresponding densitometric analysis clearly shows that treatment of NP cells with ML176 for up to 24 hours has no apparent effect on nuclear levels of HIF-1α under hypoxic conditions (Figure 5D, 5E).

### BMAL1 and RORα inhibition results in decreased expression of select HIF-1 target genes

**Figure 6:**
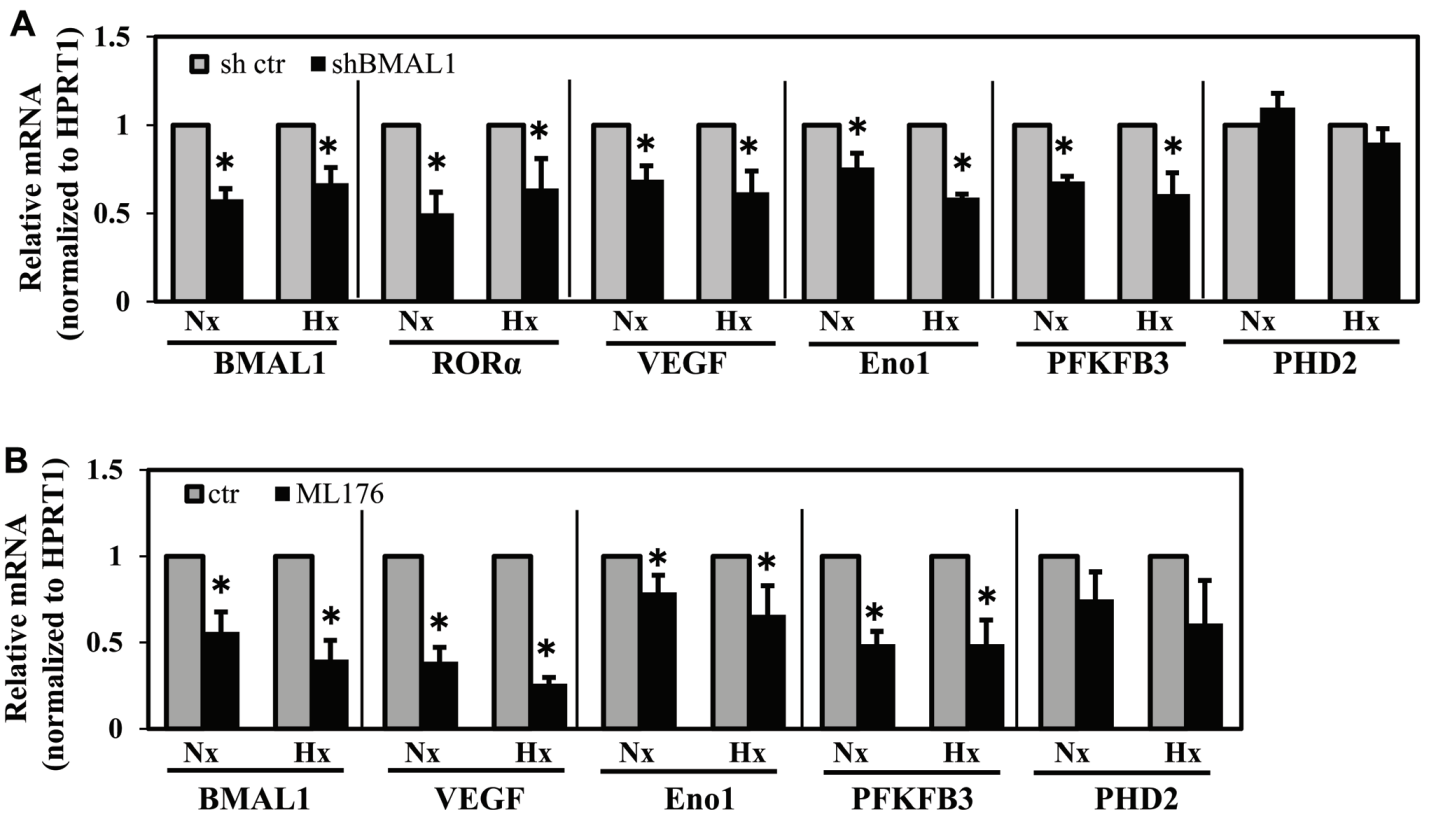
BMAL1 and RORα inhibition results in decreased expression of select HIF-1 target genes **A**.-**B**. qRT-PCR analysis of mRNA expression of select HIF-1 target genes following stable silencing of BMAL1 (A) or pharmacological inhibition of RORα activity by ML176 (10 μM) treatment. Knockdown of BMAL1 resulted in significant decrease in VEGF-A, enolase 1 (Eno1), PFKFB3 as well as RORα under both normoxia and hypoxia. B. NP cells treated with RORα inhibitor showed decreased mRNA expression of BMAL1, VEGF, PFKFB3 and Eno1. Expression of PHD2, another known HIF-1 target gene, remained unaffected in BMAL1 silenced and ML176 treated NP cells. Data is represented as mean ± SE. n=5, *p<0.1.

To confirm that suppression of BMAL1 and RORα affects HIF target gene expression, we measured mRNA expression of select HIF-1 target genes following stable silencing of BMAL1 and pharmacological inhibition of RORα activity. qRT-PCR analysis showed that knockdown of BMAL1 resulted in a significant decrease in VEGF-A, enolase 1 (Eno1), PFKFB3 and RORα under both hypoxia and normoxia (Figure 6A). Similarly, treatment of NP cells with an RORα inhibitor resulted in decreased mRNA expression of BMAL1, VEGF, PFKFB3 and Eno1 under both hypoxia and normoxia (Figure 6B). Interestingly, however, the expression of other known HIF-1 target genes, PHD2 and PHD3 (not shown), remained unaffected in BMAL1 silenced and ML176 treated NP cells.

### BMAL1 knockout mice evidence compromised disc height and vertebral bone parameters

**Figure 7:**
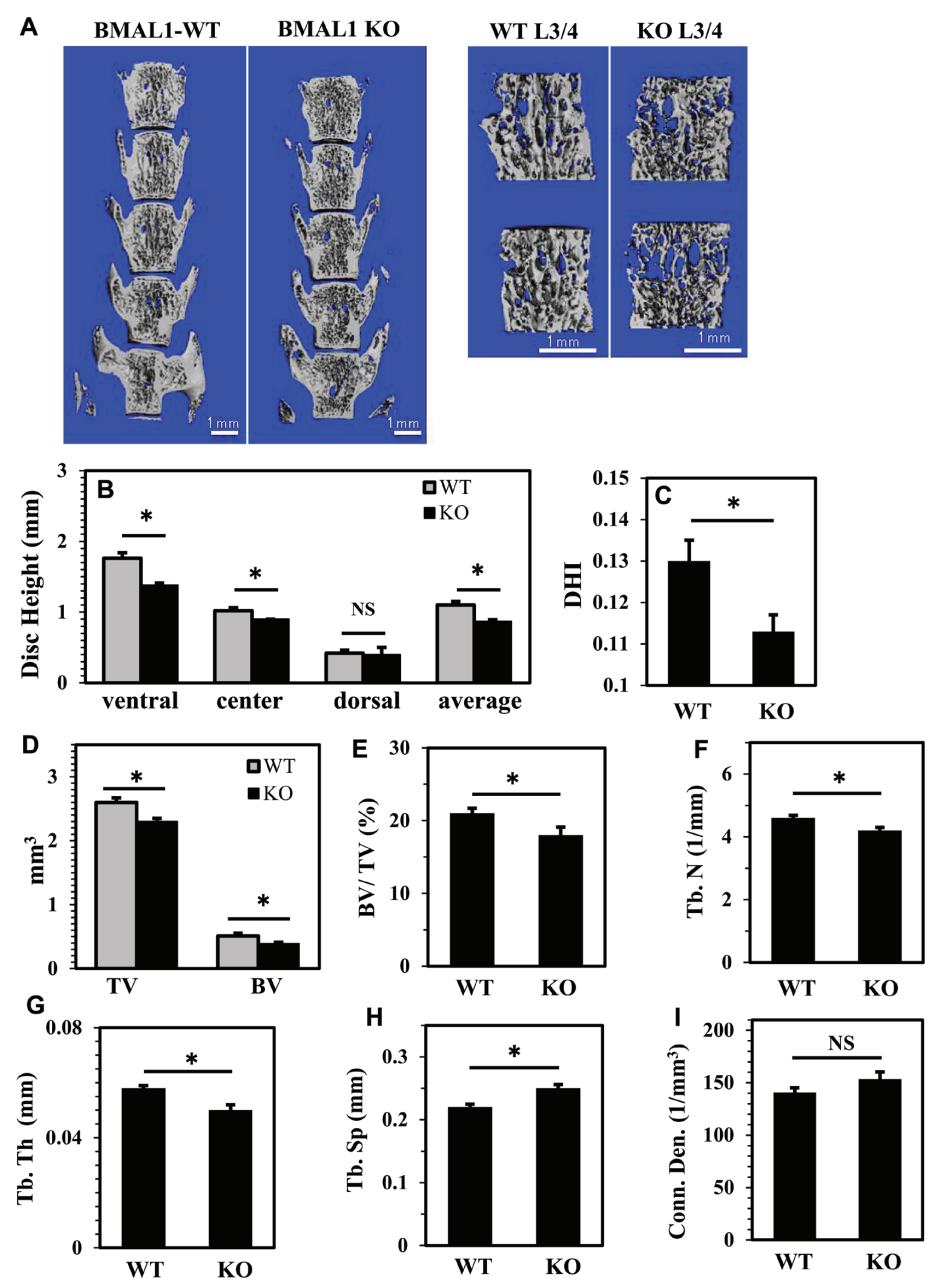
BMAL1 knockout mice (KO) evidence compromised disc height and vertebral bone health **A**. Representative μCT scans of lumbar spine (L1-L5) of a wild type and a KO littermate (10 week). Left panels show a coronal cut-plane through 3D reconstructions of the full lumbar segments of a WT and BMAL1 KO animal, showing changes in DHI. The right panels show a representative 3D reconstruction of ROI chosen for the bone trabecular morphometric analysis of each vertebral body showing changes in trabecular bone. The ROI contour only the outer boundary of the trabecular bone, excluding the cortical bone. **B**., **C**. The average of three disc height measurements (ventral, center and dorsal) (B) and disc height index (DHI) (C) were significantly lower in BMAL1-KO mice. **D**.-**H**. BMAL1 KO animals showed significant decreases in trabecular bone volume (BV) and total volume (TV) (D), bone volume fraction (BV/TV) (E), trabecular number (Tb.N) (F), and trabecular thickness (Tb. Th) (G), and a significant increase in trabecular separation (Tb. Sp) (H). I. Connectivity density (Conn. dens) was unaffected in KO animals. Data is represented as mean ± SE. n = 4 animals/genotype, 5 lumber vertebrae were measured/animal, *p<0.05.

**Figure 8:**
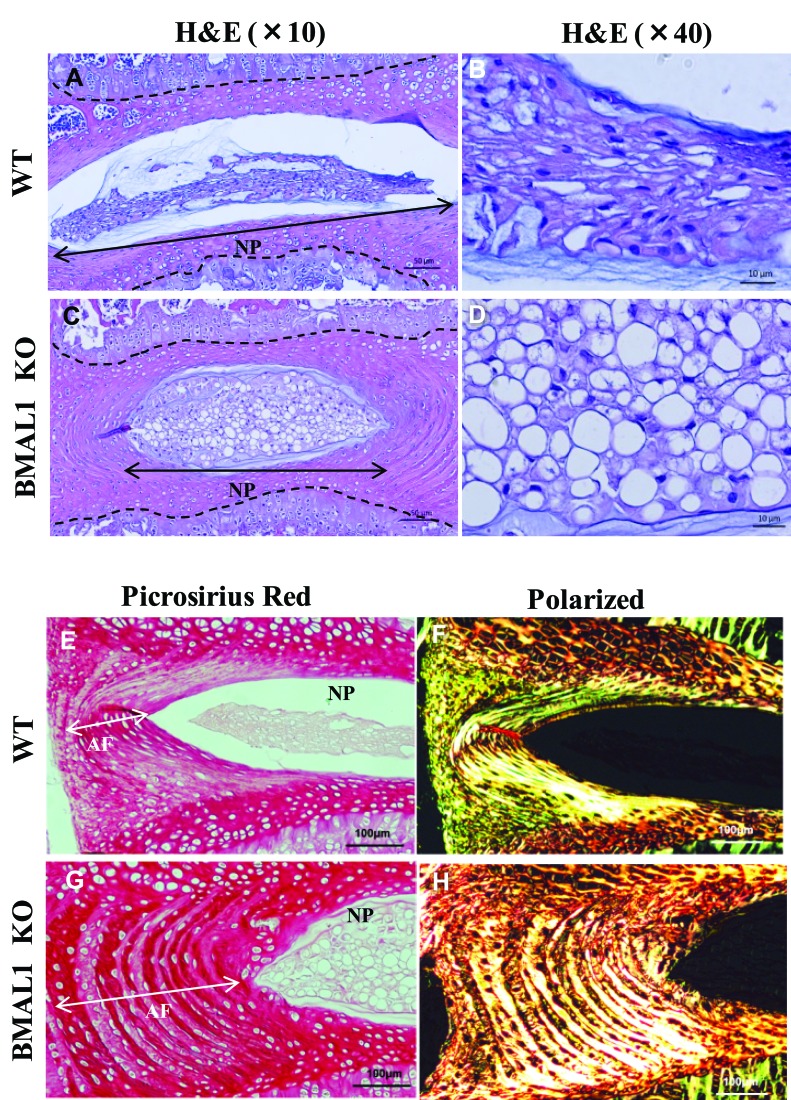
BMAL1 null mice shows altered disc morphology **A**.-**D**. Representative images of H&E and alcian blue staining of sagittal sections of lumbar disc from WT and KO mice. Compared to that of WT animals (A, B) NP tissue in null animals show decreased accumulation of matrix (lower matrix-to-cell ratio) (C, D), Scale bars: A, C: 50 μm, B, D: 10 μm. Disc height on images is outlined with dotted line. **E**.-**H**. Representative image of Picosirius red staining of WT and KO under bright field (E, G) and polarized light (F, H). Annulus fibrosus (AF) tissue in KO animals showed hyperplasia and increased thickening (white arrow). Scale bars: 100 μm.

Since BMAL1 affected HIF-1 activity in NP cells, and HIF-1 is necessary for proper functioning of the intervertebral disc, we wanted to investigate the effects of BMAL1 loss on the phenotype of post-natal spines. We analyzed the lumbar disc height of BMAL1 knockout (KO) mice (10 week old) and their wild type littermates using micro-computed tomography (μCT) (Figure 7A). Parameters of disc height and vertebral bone were measured and averaged from the lumbar spine (L1-L5). The average of three disc height measurements (ventral, center and dorsal) and disc height index (DHI) were significantly lower in BMAL1-KO than BMAL1-WT mice (Figure 7B, 7C), suggesting that *in vivo* loss of BMAL1 results in thinner discs. We also investigated the effects of BMAL1 loss on vertebral bone health. BMAL1-KO mice showed significant decreases in trabecular bone volume (BV), total volume (TV), bone volume fraction (BV/TV), trabecular number (Tb.N), and trabecular thickness (Tb. Th), and a significant increase in trabecular separation (Tb. Sp) (Figure 7D-7H). However, the connectivity density (Conn. dens) was not significantly different between WT and KO animals (Figure 7I). These results indicate that the vertebral bodies, in addition to the intervertebral discs, of BMAL1-KO animals are smaller than wild type and that the bone quality is inferior to the wild type animals. Noteworthy, loss of BMAL1 has a stronger effect on discs compared to the vertebral bodies, because the disc height index is significantly reduced in KO animals (Figure 7B). In addition, we evaluated the histological appearance of NP and lamellar structures of the annulus fibrosus (AF) (Figure 8A-8H). Not surprisingly, NP of BMAL1 null mice did not phenocopy that of NP-specific HIF-1α-deficient mice, in that NP cells of BMAL1 null mice remained viable post-natally [13]. However, analysis showed that BMAL1-KO animals have lower matrix-to-cell ratio (Figure 8C, 8D, and 8G), whereas in WT animals this ratio was higher (Figure 8A, 8B and 8E). Moreover, AF tissue in BMAL1-KO (Figure 8G, 8H) evidenced hyperplasia (Figure 8E, 8F) though the fiber orientation did not show obvious differences between WT and KO.

## DISCUSSION

BMAL1 (brain and muscle ARNT-like1), CLOCK and HIF-α are basic helix-loop-helix-PAS (bHLH-PAS) transcriptional factors that share similarities in their bHLH and PAS dimerization domains [22, 30]. Canonically, BMAL1 acts through heterodimerization with CLOCK, and binding to E-box motifs -CACGTG in target gene promoters [17, 18], while HIF-α binds to its partner HIF-1β/ARNT to transcribe targets that allow cellular adaptation to hypoxia. A few reports have described a non-canonical function of BMAL1, dimerizing with HIF-1α under hypoxia, and activating transcription through binding to HRE motifs [23–25]. However, this hypoxia and HIF-dependent role of BMAL1 seems to be cell-type specific [31]. Since HIF-1α is necessary for NP cell survival in the hypoxic niche [13], we sought to investigate the potential role of the circadian master regulator BMAL1 in controlling HIF-1 function in the intervertebral disc.

In our findings, BMAL1 overexpression alone did not change HRE activity. Noteworthy, co-transfection of BMAL1 with HIF-1α increased HRE activity compared with HIF-1α expression alone. This suggests that BMAL1 in NP cells can cooperate with HIF-1α to activate HRE mediated transcription. Interestingly, our evaluation of HIF-TAD domains indicated that BMAL1 overexpression did not modulate recruitment of other co-factors by HIFs. Surprisingly, immunoprecipitation results showed neither BMAL1 nor CLOCK bound to HIF-1α, suggesting either lack of binding or a very transient nature of their interaction. These results are also in agreement with a report by Ghorbrl et al. demonstrating that BMAL1, CLOCK and HIF-1α cooperatively affected transcriptional regulation of vasopressin without heterodimerization [25]. It is therefore not unreasonable to assume that in NP cells BMAL1 activates HIF-1α function without direct dimerization.

To further assess the function of the BMAL1-HIF axis in NP, we evaluated HIF activity in BMAL1-silenced NP cells. Downregulation of HRE activity in BMAL1-silenced NP cells demonstrates that BMAL1 controls HIF-1α activity. Importantly, when BMAL1 expression was suppressed, VEGF-A, Eno1 and PFKFB3 mRNA expression were downregulated. These three HIF-1 targets have been reported to contain E-box motif in their promoters and are sensitive to BMAL1 mediated regulation. A direct BMAL1 interaction with the E-Box has also been demonstrated in the case of VEGF-A promoter [32–35]. Noteworthy, the relationship between E-box motif and HRE sequence has been reported; although HIF-1 binding does not usually occur at the palindromic E-box, high percentage of rat, mouse and human gene promoters with functionally validated HREs overlap with predicted E-boxes [36–38]. These reports implicate that binding of BMAL1:CLOCK to the E-box sequences in gene promoters may affect HIF-1α recruitment and activity at nearby or overlapping HRE sites, effectively regulating its target gene expression.

Furthermore, we investigated the role of RORα in NP cells. It has been well known that RORα is an important regulator of BMAL1, and they regulate each other's expression via a feedback loop [15, 19, 20]. Our results that show decreased BMAL1 mRNA in RORα inhibitor treated cells and suppressed RORα protein and mRNA in BMAL1-silenced NP cells clearly support an inter-relationship between BMAL1 and RORα. Importantly, similar to BMAL1, RORα promoted HIF-1α-dependent HRE activity under hypoxia. A previous study with NIH3T3 cells reported that the DNA-binding domain of RORα directly interacted with the inhibitory domain of HIF-1α, increasing its stability [26]. However, our immunoprecipitation experiments did not support an overt RORα-HIF-1α interaction in NP cells if any, suggesting a possible cell-type specific characteristic. Together these results indicate that RORα influences HIF-1α activity plausibly without a direct protein-protein interaction between them. Interestingly, inhibition of RORα activity by ML176 resulted in a decreased HIF-1α-TAD activity as well as mRNA expression of VEGF-A, PFKFB3, Eno1, and BMAL1. These findings indicate that RORα may affect HIF-1 activity and target gene expression in NP cells through modulating co-factor recruitment. Since BMAL1 and RORα coregulate each other's expression, they are likely to influence HIF-1α in a cooperative fashion.

Our studies show for the first time that BMAL1 and RORα, two important molecules in circadian rhythm machinery, are involved in the regulation of HIF-1α transcriptional function in NP cells through an indirect mechanism. In fact, the evaluation of BMAL1 KO mice demonstrated that BMAL1 is important for maintenance of discs and vertebral health. Both disc height and vertebral volume were smaller in BMAL1 KO mice compared to WT. Importantly, DHI of BMAL1 KO was also decreased, indicating a more crucial role for BMAL1 in maintenance of disc health compared to vertebral bodies. These results complimented the studies that showed early aging in other tissues from BMAL1 deficient mice [39]. It was also not surprising to see that NP phenotype of BMAL1 null mice did not phenocopy that of NP-specific HIF-1α-deficient mice, where NP cells die postnatally [13]. Since BMAL1 co-regulated only a subset of HIF-1 target genes in NP cells, and because it is one of many regulators of HIF-1α function, lack of NP cell death is to be expected. Thus, this observation suggests that BMAL1 is dispensable for survival of notochordal NP. Still, histological studies of BMAL1 null mice showed decreased matrix-to-cell ratio in post natal NP. This implicated a possible delay in maturation and decreased secretion of proteoglycan rich matrix by notochordal cells that give rise to adult NP [40]. Whether this NP phenotype is due to modulation of HIF-1 transcriptional activity controlling cell proliferation and differentiation in NP cells [41] or due to BMAL1 function independent of HIF-1 remains to be seen. In contrast to the effects on NP compartment, AF tissue in BMAL1 null mice evidenced hyperplasia, suggesting that the effects of BMAL1 deletion are tissue specific. Relevant to this discussion, in a recent study Numaguchi et al. showed that rats exposed to passive cigarette smoking for 8 weeks showed a significant alteration in many clock-related genes in both AF/CEP and NP tissues of the intervertebral disc [42]. Importantly, in discs these clock genes showed expression patterns consistent with a circadian rhythm, with several genes including Per1, Per2, Cry1, Nr1d1 and Npas2, clearly evidencing a phase shift of anywhere between 3 to 9 h due to smoking. Significantly, BMAL1 and another clock gene, Dbp, demonstrated complete abrogation of oscillation in the NP in smoke exposed animals [42]. Since smoking is considered an important risk factor for disc degeneration, and has been shown to promote NP cell dysfunction and matrix deposition [43, 44], it is plausible that some of the pathological changes seen in disc in these smoke exposed animals may be attributed to altered clock gene activity. Taken together, our results suggest that BMAL1 and RORα regulate HIF-1 activity in NP cells and play an important role in overall adaptation of NP cells to their hypoxic niche, and their dysregulation affects normal tissue homeostasis and function. Further investigations on the contribution of BMAL1 in progression of disc degeneration are under way.

## MATERIALS AND METHODS

### Plasmids and reagents

Plasmids were kindly provided by Dr. Taku Saito, University of Tokyo, Japan (HA-ARNT, -ARNT2, and -BMAL1) [27], Dr. Ren Xu, University of Kentucky, Lexington (pCDH1-RORα-flag) [45], and Dr. Vijay K. Yechoor, Baylor College of Medicine, Houston (Lentiviral shBMAL1) [46]. HIF-1α-C-TAD, aa740-826; HIF-1α-N-TAD, aa 530-778; and HIF-2α-TAD, aa 819–870 were provided by Dr. Nianli Sang, Drexel University, Philadelphia, PA. pFR-Luc (Stratagene) reporter contains the firefly luciferase gene. psPAX2 (# 12260) and pMD2G (#12259) developed by Dr. Didier Trono and HRE-Luc (#26731) were obtained from Addgene's repository. For internal transfection control, vector pRL-TK (Promega) containing Renilla reniformis luciferase gene was used. RORα inverse agonist ML176 was purchased from Cayman Chemical (Michigan). This selective inverse agonist competitively blocks the binding of 25-hydroxycholesterol to the ligand-binding domain of RORα and inhibits transactivation function. Spines of BMAL1^−/−^ mice (9-10 weeks old) were kindly provided by Dr. Vijay Yechoor [46]. These mice were generated by breeding of Bmal1+/− on a C57Bl/6J background and maintained under 12:12 Light/Dark cycles, with lights on at 7 AM—Zeitgeber Time 0 (ZT-0) under ad lib access to food and water.

### Isolation of NP cells, cell treatments and hypoxic culture

Rat (Sprague-Dawley, 10 week old) or mouse (C57Bl/6J, 10 week old) NP cells were isolated using a method reported earlier by Risbud et al. [8]. Animals were maintained under 12:12 Light/Dark cycles with ad lib access to food and water. Cells were maintained in Dulbecco's modified Eagle's medium (DMEM) and 10% fetal bovine serum (FBS) supplemented with antibiotics, and cultured in a Hypoxia Work Station (Invivo2 300, Ruskinn, UK) with a mixture of 1% O2, 5% CO2 and 94% N2 for 8-72 h.

### RNA isolation and Real Time RT-PCR Analysis

For analysis of BMAL1 and RORa in disc compartments, NP and AF tissues were isolated from adult Sprague-Dawley rats and immediately incubated in RNAlater (Thermofisher, AM7021) at 4°C. NP tissue was transferred to lysis buffer and homogenized with a Pellet Pestle Motor (Sigma Aldrich, Z359971) for 1 minute. AF tissue was frozen in liquid nitrogen and pulverized with a BioPulverizer (BioSpec). Pulverized AF tissue was transferred to TRIzol Reagent (Lifetechnologies) and DNA-RNA was isolated according to Lifetechnologies protocol and a DNA-free kit (Lifetechnologies). Total RNA was extracted from NP cells and NP tissue lysates using RNAeasy mini columns (Qiagen). Before elution from the column, RNA was treated with RNase free DNase I (Qiagen). The purified, DNA-free RNA was converted to cDNA using EcoDry™ Premix (Clontech). Template cDNA and gene-specific primers were added to Power SYBR Green master mix (Applied Biosystems) and mRNA expression was quantified using the Step One Plus Real-time PCR System (Applied Biosystems). HPRT1 was used to normalize gene expression. Melting curves were analyzed to verify the specificity of the RT-PCR and the absence of primer dimer formation.

### Transfections and dual luciferase assay

Cells were transferred to 48-well plates (2×10^4^ cells/well) one day pre-transfection. Cells were transfected with 175 ng HRE luciferase reporter with or without 25 ng of HA-HIF-1α and 0-150 ng of ARNT or ARNT2, or BMAL1 or RORα or empty backbone plasmids and 150 ng pRL-Tk, and reporter activities measured following culture under normoxia or hypoxia for 24 h. To measure effect of RORα inhibition on HIF-1 activity, cells were transfected with HRE reporter and activity measure after treatment with 0-10 μM ML176 for 24 h. To measure effect on HIF transactivation, cells were cotransfected with HIF-α-TAD plasmids (25 ng each) with or without 150 ng of BMAL1, and activity measured following normoxic or hypoxic culture for 24 h. TAD activity was also measured by treating cells with RORα inhibitor ML176 (10 μM) for 24 h. In all experiments, plasmids were premixed with the transfection reagent, LipofectAMINE2000 (Invitrogen). The next day, cells were harvested and Dual-Luciferase™ reporter assay system (Promega) was used for measurements of firefly and Renilla luciferase activities using a luminometer (TD-20/20, Turner Designs CA).

### Protein Extraction, nuclear fractionation and Western blotting and Immunoprecipitation

Cells were immediately placed on ice and washed with ice-cold PBS. All wash buffers and the final resuspension buffer included 1x protease inhibitor mixture (GE Healthcare), NaCl (150 μM), β-glycerophosphate (62.5 mM), DTT (0.1 μM), NaF (5 mM), and Na_3_VO_4_ (200 μM). When needed, CelLytic NuCLEAR extraction kit (Sigma Aldrich) was used to generate nuclear proteins. Immunoprecipitation was performed using Protein A/G PLUS-Agarose beads (SantaCruz) following standard protocol. Proteins were resolved on 8–12% SDS-polyacrylamide gels and transferred by electroblotting to PVDF membranes (Bio-Rad). The membranes were blocked with 5% nonfat dry milk in TBST (50 mM Tris pH 7.6 with 0.1% Tween 20) and incubated overnight at 4°C in 5% nonfatdry milk in TBST with antibody. Immunolabeling was detected using the ECL reagent (Amersham Biosciences). Relative expression levels were determined by quantitative densitometric analysis using one-dimensional image analysis software (GE Healthsciences). Antibodies used were anti-BMAL1 (#14020, Cell Signaling), anti-CLOCK (#5157, Cell Signaling; #3517, Abcam), anti-RORα (#NBP1-52813, Novus), anti-HIF-1α (#MAB1536, R&D Systems; #ab2185, Abcam), anti-HIF-1β/ARNT (#5537, Cell Signaling), anti-GAPDH (#NB300-221SS, Novus) and anti-Lamin A/C (#2032, Cell Signaling).

### Lentiviral production and transduction

HEK 293T cells were seeded in 10-cm plates (5×10^6^ cells/plate) in DMEM with 10% heat-inactivated FBS one day before transfection. Cells were transfected with 9 μg of shBMAL1 or pGIPZ plasmids along with 6 μg psPAX2 and 3 μg pMD2G using Calcium phosphate transfection kit (CalPhos, Clontech). After 16 h, transfection medium were removed and replaced with DMEM with 10% heat-inactivated FBS. Lentiviral particles were harvested at 48 and 60 h post-transfection. NP cells in 10 cm plates were transduced with 8 ml of medium containing viral particles with 6 μg/ml Polybrene. After 24 h, viral media was removed and replaced with DMEM with 10% FBS. 5 days after viral transduction, cells were used for protein and mRNA evaluation.

### Micro-computed tomography analysis of BMAL1 knockout mouse spine

Micro-computed tomography scans (MicroCT40, SCANCO Medical, Switzerland) were performed on spines of BMAL1^−/−^ and wild type mice (n=4/genotype) fixed with 4% PFA. Segments of lumbar spine incorporating L1-L5 were scanned with an energy of 70 kVp, a current of 114 mA, and a 200-ms integration time producing a resolution of 16 mm^3^ voxel size. The cross sectional scans were analyzed to quantify changes in trabecular bone microarchitecture by first drawing regions of interest (ROI) that contour the outer boundary of the trabecular bone, excluding the cortical bone. The ROI were then compiled into 3D data sets using a Gaussian filter (σ = 1.0, support = 1) to reduce noise, and converted to binary images with a fixed grey-scale threshold of 200. The 3D data sets were assessed for the following parameters using software supplied by the system manufacturer: trabecular bone volume (BV), total volume (TV), bone volume ratio (BV/TV), trabecular thickness (Tb. Th), trabecular number (Tb.N) trabecular separation (Tb. Sp), connectivity density (Conn.dens.) and disc height between each vertebra. Disc height was determined by measurements made in three areas of the disc space: on the ventral, central and dorsal side, and an average of these values. Disc height index (DHI) was calculated by dividing average disc height by height of adjacent vertebral bodies. All heights were calculated by multiplying the number of cross sectional slices spanning the structure of interest by the known thickness of each slice.

### Immunohistological analysis

Following microCT spines were embedded in paraffin. Sagittal sections, 6-8 μm in thickness, were deparaffinized in xylene, rehydrated through graded ethanol and stained with alcian blue, eosin, and hematoxylin, or Picrosirius Red Stain kit ^TM^ (Polysciences, Inc.). For localizing BMAL1, sections of rat discs were incubated with the anti-BMAL1 antibodies (#AB 4140, Millipore) in 5% goat serum in PBS at 4°C overnight. After thoroughly washing the sections, the bound primary antibodies were incubated with Alexa fluor-594 conjugated anti rabbit secondary antibody (Invitrogen) for 1 h at room temperature. Sections were visualized using a fluorescence microscope (Axio Imager 2, Zeiss) or a polarizing microscope (Eclipse LV100 POL, Nikon).

### Statistical analysis

All measurements were performed in triplicate, and data are presented as mean ± S.E. Differences between groups were analyzed by the Student's *t* test and one-way ANOVA; p<0.05.
